# Double-layered suturing using a reopenable-clip over-the-line method with a 20-mm reopenable clip to close a large gastric mucosal defect

**DOI:** 10.1055/a-2739-2220

**Published:** 2025-11-28

**Authors:** Tatsuma Nomura, Yoshiaki Isono, Takanobu Mitani, Yuto Ikadai, Takanori Takenaka, Hiroaki Kumazawa, Katsumi Mukai

**Affiliations:** 1 Department of Gastroenterology, Suzuka General Hospital, Suzuka, Japan; 2 Department of Endoscopy Center, Suzuka General Hospital, Suzuka, Japan

Closing large defects left after endoscopic submucosal dissection (ESD) of the upper gastric body is difficult because of the thick muscle layer. We recently developed the reopenable-clip over-the-line method (ROLM) to assist such closure procedures 1 2 3 . Furthermore, a 20-mm reopenable clip (LOCKADO clip; 20 mm; Micro-Tech (Nanjing) Co., Ltd, Nanjing, China) with a large opening width has recently become available in Japan 4 .

We have now also explored a double-layered ROLM (DL-ROLM) approach that achieves complete closure using a large clip combined with double-layered suturing ( Fig. 1 ).

**Fig. 1:**
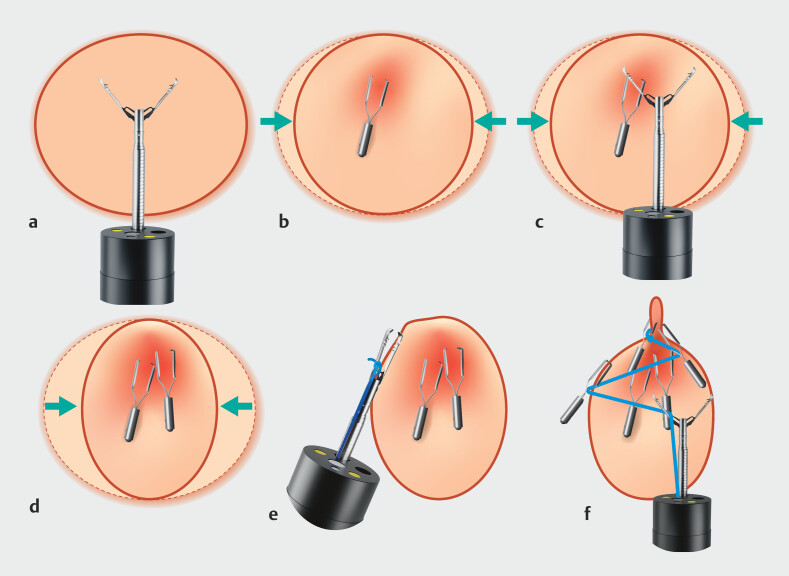
Schematic representation of the double-layered suturing with a reopenable clip over-the-line method (DL-ROLM), using a 20-mm reopenable clip. **a** Circular ESD defect and the 20-mm reopenable clip with small protrusions to prevent slippage. **b** The clip is placed such that it grips the muscle layer of the ulcer floor. **c** and **d** The muscle layer that grasps the adjacent muscle layer creates a fold that grasps the defect’s edge. **e** and **f** Transforming the defect into an oval shape allows its edges to be brought closer together. This facilitates a simpler closure using the reopenable clip-over-the-line method (ROLM).

The wide, reopenable clip features small protrusions to prevent slippage, allowing it to grab more tissue than conventional clips. Its reopenable function facilitates substantial muscle grasping.

We recently treated a patient with a large post-ESD defect left in the stomach’s upper lesser curvature ( Fig. 2 and Video 1 ). Because of significant muscle layer damage we observed during the ESD, we closed the defect entirely, using DL-ROLM. We grasped a large portion of the muscle on the floor of the ulcer and placed the reopenable clip 5 . A second clip was then placed to grasp the folds created by the initial clip, as well as the musculature on the ulcer floor. Additional clips were placed similarly to grasp the regional muscles. Together, the clips held the defect in an oval shape, facilitating further closure using the ROLM approach. Threaded clips were then placed along the edges of the defect. The clips with lines threaded through their teeth were placed on the contralateral edge of the defect. We confirmed endoscopically that the clip had not been buried. The repeated placement of clips with lines threaded through the tooth was then able to completely close the large defect, and the patient was discharged without any adverse events.

**Fig. 2:**
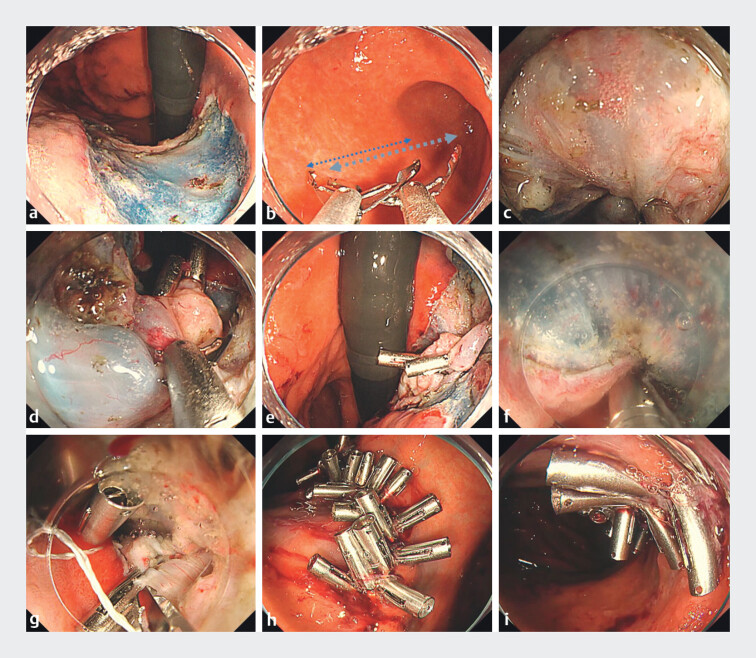
DL-ROLM performed with a 20-mm reopenable clip for closing a large mucosal defect left after gastric ESD. **a** A large gastric defect extending from the wall of the lesser curvature’s upper body to the anterior wall. **b** A traditional 16-mm reopenable clip for this application (left), vs the newly available 20-mm reopenable clip (right). **c** The 20-mm reopenable clip was used to grasp the muscle at the ulcer floor. **d** and **e** Folds in the muscle layer were created by repeated applications of the 20-mm reopenable clip, and the musculature was grasped using the clip. This brought the edges of the defect closer, thereby facilitating full closure using additional clips. **f** The ROLM applied, under saline immersion. **g** and **f** ROLM facilitated defect closure without embedding the clips into the muscularis layer to grasp the muscle within the defect. **h** and **i** Complete closure of the large gastric defect left after gastric ESD.

The DL-ROLM procedure using a 20-mm reopenable clip to close a large mucosal defect left after gastric ESD.Video 1

By folding the muscle over a large mucosal defect, DL-ROLM facilitates ROLM and a more secure closure, potentially extending the duration over which closure is maintained.

Endoscopy_UCTN_Code_TTT_1AO_2AO

## Notes

Endoscopy E-Videos https://eref.thieme.de/e-videos
*E-Videos* is an open access online section of the journal *Endoscopy* , reporting on interesting cases and new techniques in gastroenterological endoscopy. All papers include a high-quality video and are published with a Creative Commons CC-BY license. Endoscopy E-Videos qualify for HINARI discounts and waivers and eligibility is automatically checked during the submission process. We grant 100% waivers to articles whose corresponding authors are based in Group A countries and 50% waivers to those who are based in Group B countries as classified by Research4Life (see: https://www.research4life.org/access/eligibility/ ). This section has its own submission website at https://mc.manuscriptcentral.com/e-videos .
